# Quality-of-life outcomes and unmet needs between ileal conduit and orthotopic ileal neobladder after radical cystectomy in a Chinese population: a 2-to-1 matched-pair analysis

**DOI:** 10.1186/s12894-015-0113-7

**Published:** 2015-11-27

**Authors:** Yi Huang, Xiuwu Pan, Qiwei Zhou, Hai Huang, Lin Li, Xingang Cui, Guodong Wang, Ren Jizhong, Lei Yin, Danfeng Xu, Yi Hong

**Affiliations:** Department of Urinary Surgery of Changzheng Hospital, Second Military Medical University, No. 415, Fengyang Road, Huangpu District, Shanghai 200003 China; Department of Urinary Surgery of Navy Hospital of Xiamen, No. 23, Zhenhai Road, Siming District, Xiamen 361000 China; Department of Urinary Surgery of Third Affiliated Hospital, Second Military Medical University, No. 700, Moyu Road, Jiading District, Shanghai 201805 China; Department of Urinary Surgery of No. 313 Hospital of PLA, No. 50, Haibinnan Road, Longgang District, Huludao City, Liaoning 125000 China; Department of Stomatology of Changzheng Hospital, Second Military Medical University, Shanghai, China

**Keywords:** Bladder cancer, Laparoscopy, Radical cystectomy, Orthotopic Ileal, Neobladder, Ileal conduit, Quality-of-life

## Abstract

**Background:**

Health-related quality-of-life (HRQoL) is an important consideration after radical cystectomy (RC). Lack of effective ways to assess HRQoL after RC and unawareness of disease-specific problems related to ileal conduit (IC) and orthotopic ileal neobladder (OIN) are serious problems. The present study was to evaluate and compare morbidity and HRQoL between IC and OIN after RC, and examine their unmet needs in the two groups.

**Methods:**

A retrospective analysis was made of 294 patients treated with RC in our hospital between 2007 and 2013. Matched pair analysis was used to determine the patients of IC and OIN groups. Patient HRQoL between IC and OIN groups was assessed using the bladder-specific bladder cancer index (BCI) and European Organization for Research and Treatment of Cancer Body Image scale (BIS) questionnaires. Unmet information of patients undergoing these two urinary diversions was recorded through individual interviews.

**Results:**

Of the 117 included patients, 39 patients were treated with OIN and the other 78 matched patients with IC as controls for matched pair analysis. There was no significant difference in baseline characteristics between the two groups. OIN patients showed significantly better BIS scores in terms of HRQoL outcomes after RC at a short-term (<1 year) follow-up level, but there was no significant difference at a long-term (>1 year) follow-up level between the two groups. Interestingly, urinary bother (UB) and urinary function (UF) were poor in OIN patients at the one-year follow-up level, but there was no significant difference in UB between the two groups at the long term follow-up level. Unmet needs analysis showed that OIN patients had a more positive attitude towards treatment and participated in physical and social activities more positively, although they may have more urine leakage problems.

**Conclusions:**

The mean BIS score in OIN group patients was significantly better than that in IC group patients at the one-year follow-up level, but there was no significant difference at the long-term follow-up level. Due attention should be paid to some particular unmet needs in individual patients in managing the two UD modalities.

**Electronic supplementary material:**

The online version of this article (doi:10.1186/s12894-015-0113-7) contains supplementary material, which is available to authorized users.

## Background

Radical cystectomy (RC) with bilateral pelvic lymphadenectomy is the standard treatment for muscle-invasive and high-risk non-muscle-invasive urothelial carcinoma of the bladder (UCB) [1]. There are numerous choices for urinary diversion (UD) after RC. Ileal conduit (IC) continues to be the most common form of UD, while orthotopic ileal neobladder (OIN) is the preferred continent UD in some patients owing to its functional and psychological advantages [2, 3], although it is contraindicated for patients with intraurethral tumors and urethral stricture.

In the past decade, health-related quality-of-life (HRQoL) is an important consideration after RC, because this traumatic event is often associated with significant changes in body image, urinary and sexual functions, interpersonal relationships, and psychosocial stress outcomes [4]. Although there is no convincing evidence to support the conclusion that OIN is superior to IC [5], most patients are likely to choose OIN at the individual level despite the informed risk of problematic orthotipic voiding.

Given the controversies over the choice of different UD options partly due to the lack of effective ways to assess HRQoL after RC and unawareness of disease-specific problems related to IC and OIN in most Chinese patients, the present study retrospectively compared the discrepancies in the HRQoL of patients treated with IC or OIN, in an attempt to better assess the two different UD by combined use of BIS and BCI questionnaires during the follow-up periods. In addition, specific unmet needs in patients of the two groups were also clarified.

## Methods

### Patient selection

Recruited in this study were patients who underwent RC at the Department of Urology of Shanghai Changzheng Hospital (Shanghai, China) between January 2007 and December 2013. Inclusion criteria were patients who underwent RC with OIN or IC and were able to fully understand the questionnaires and fill out the questionnaire forms. Exclusion criteria were 1) patients who had psychiatric disorders, histories of alcohol or substance abuse, cognitive morbidity such as Alzheimer’s disease, or additional oncological disease; 2) patients whose follow-up duration was less than one year; and 3) patients who were lost to follow-up or experienced recurrences or died during the one-year follow-up period. Finally, 205 IC patients and 89 OIN patients were included in this study for analysis. This study was approved by the ethics board of Shanghai Changzheng Hospital in accordance with the Declaration of Helsinki, and all patients provided informed consent.

### Surgical procedures

Most patients in our center underwent laparoscopic radical cystectomy (LRC) after RC, using the OIN reconstruction technique described by Hautmann et al. [6]. Contraindications for OIN were ASA score > 3, severe cardiac insufficiency, decompensated pulmonary function, impaired renal function (serum creatinine >2 mg/dL), the presence of intraurethral tumors and/or urethral stricture, a history of previous bowel resection, abnormal abdominal straining and extensive muscle-invasive UCB. The patient who has these contraindications for OIN chose IC reconstruction. The IC was constructed in a standard fashion by using the minimum amount of the ileum. However, some patients who did not have these contraindications for OIN chose between IC and OIN reconstruction after impartial counselling. Standard pelvic lymph node dissection (PLND) was performed in all patients.

### Baseline patient characteristics

Data regarding preoperative, perioperative and pathologic characteristics were collected. Preoperative variables included age, sex, body mass index (BMI), ASA classification, comorbid conditions (previous abdominal/pelvic surgery, other malignancy, cardiovascular disease, pulmonary disease, hypertension and diabetes), and the smoking status. Perioperative variables were operative time (OT), estimated blood loss (EBL), the number of lymph nodes removed, transfusion, conversion to open surgery, and short-term complications classified according to the modified Clavien classification system [7]. Pathologic characteristics were histopathologic tumor type, grade and stage defined according to the TNM classification and the WHO System 2004.

### Outcome evaluation

HRQoL outcomes were measured using the BIS [8] and BCI [9] questionnaires. All the questionnaire forms were filled out by patients personally and completed preoperatively and at 6-month intervals after surgery. The BIS published by Hopwood et al. [8] for assessing body image changes in patients with cancer is generally accepted as a brief patient self-report measure in conjunction with the EORTC-QOL Study group. It contains 10 items including affective, behavioral and cognitive aspects. Each item is scored on a 0–3 scale and the overall summary scores range from 0 to 30, with higher scores representing progression of the symptoms. The BCI initially created by Gilbert et al. [9] consists of 34 items within three primary domains assessing urinary, bowel and sexual functions. All these items are responded through the 5-point Likert scale. Each primary domain contains two parts (function and bother) and are standardized to the 0–100 scale, with higher scores representing better HRQoL. The unmet need information was retrospectively collected by another interview guide design by Mohamed et al. [10].

Peri-operative outcomes (OT, EBL, the number of lymph nodes removed, transfusion and conversion to open surgery) are shown in Additional file 1: Table S5. The simple outcomes following OIN including the daytime/continence rate, ISC rate, maximum neobladder volume (mL) and postvoid residual are shown in Additional file 2: Table S6.

### Statistical analysis

Patients were selected for matched pair analysis (MPA) and blinded to outcomes. Exact matching was performed for the following primary factors potentially correlated to the poor preoperative QoL: age at operation (the allowed difference was less than 10 years), gender (needs to be identical), and ASA (needs to be identical). The matching process resulted in 117 patients involved.

All statistical analyses were performed using SAS software (v9.3, SAS Institute, Cary, NC). The results are presented as mean (range) values and percentages. The Chi-Square test and Wilcoxon rank-sum test were used to see whether there was a significant difference between the two UDs. The repeat measures of ANOVA (analysis of variance) were performed in the 1-year follow-up and the long-term follow-up periods (longer than 1 year), respectively. A P value <0.05 was considered statistically significant.

## Results

A total of 294 patients underwent RC with pre- and postoperative follow-up data available. Pair matching identified 39 OIN and 78 IC patients suitable for inclusion in this analysis. The patient demographics and clinical characteristics are shown in Table 1 and Additional file 3: Table S1.

**Table 1:** Demographics and clinical characteristics data after matched pair analysis

Characteristic	After MAP (1:2) 117		*P*
	OIN (39)	IC (78)	
Mean age, yr (range)	63.6 (51.5–76.0)	64.0 (52.0–74.8)	0.885^a^
Sex ratio (M/F)	34/5	68/10	match
Mean BMI, kg/m2 (range)	21.7 (18.7–24.4)	22.0 (18.8–25.4)	0.637^a^
ASA class, N (%)			Match
1	6 (15.4)	12 (15.4)	
2	24 (61.5)	48 (61.5)	
3	9 (23.1)	18 (23.1)	
Smoking history, N (%)	9 (23.1)	20 (25.6)	0.762^c^
Previous abdominal or pelvic surgery, N (%)	4 (10.3)	11 (14.1)	0.558^c^
Comorbidities, N (%)			
Cardiovascular disease	3 (7.7)	8 (10.3)	0.911^b^
Pulmonary disease	5 (12.8)	16 (20.5)	0.307^c^
Hypertension	9 (23.1)	28 (35.9)	0.160^c^
Diabetes	7 (17.9)	16 (20.5)	0.742^c^
Histological type, N (%)			0.890^b^
Pure TCC	36 (92.3)	74 (94.9)	
Other pathology	3 (7.7)	4 (5.1)	
Histological grade, N (%)			0.651^c^
Grade 1 and Grade 2	9 (23.1)	15 (19.2)	
Grade 3	28 (71.8)	58 (74.4)	
Pathologic T stage, N (%)			0.476^a^
Organ-confined: ≤pT2, pN0	33 (84.6)	62 (79.5)	
Non-organ-confined: pT3-pT4, pN0	6 (15.4)	14 (17.9)	
Lymph node-positive: pN+	0	2 (2.6)	
Highest grade of complication, N (%)			0.562^a^
I	4 (10.3)	8 (10.3)	
II	4 (10.3)	7 (9.0)	
III	3 (7.7)	3 (3.8)	
IV	0	0	
V	0	0	

^a^Wilcoxon rank-sum test

^b^Pearson chi-squared test with continuity correction

^c^Chi-Square

**Fig. 1:**
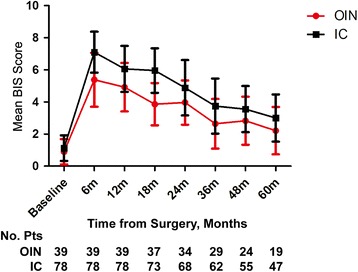
The BIS scores over time including all time point information to compare the difference between IC and OIN

**Fig. 2:**
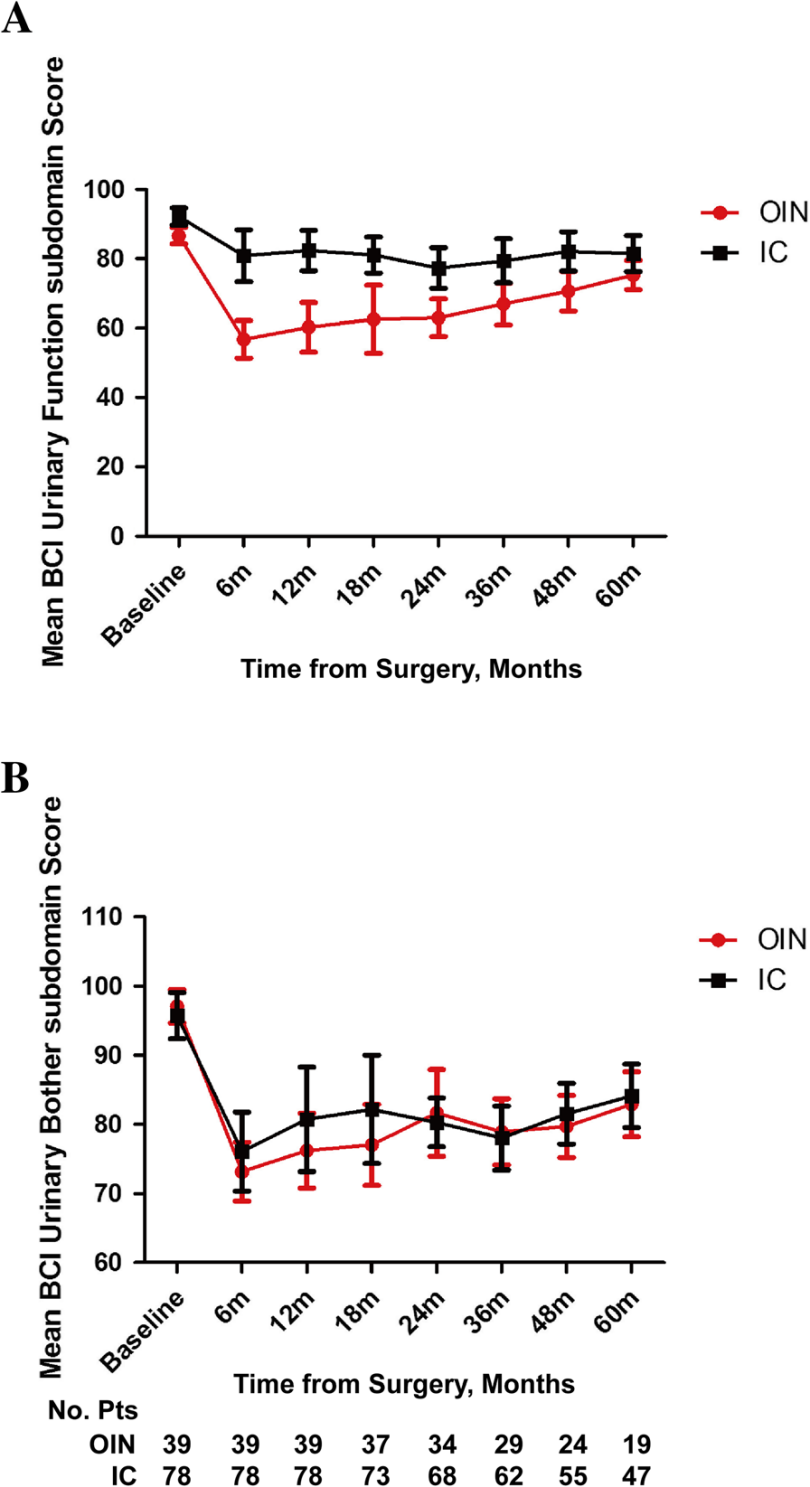
**a** The mean BCI urinary function score between IC and OIN. **b** The mean BCI urinary bother score between IC and OIN

Figure 1 presents the mean BIS score for each UD over time. The mean BIS score in IC patients was lower than that in OIN patients in the one year follow-up period (*P* = 0.003). However, there was no significant difference between the two UD types at the long-term follow-up level (*P* = 0.114), suggesting that time had a positive effect on BIS of both groups (*P* <0.001). Figure 2a shows that urinary function (UF) based on BCI score was better in IC patients than that in OIN patients at both one-year follow-up and long-term follow-up levels (*P* < 0.001 and *P* = 0.0074). Interestingly, ANOVA analysis showed that with time prolonging, UF was improved in both groups as compared with their previous state (*P* < 0.001). Figure 2b shows that urinary bother (UB) in terms of BCI score favored IC group at the one-year follow-up level (*P* = 0.004), although the two UD types showed no significant difference (*P* = 0.720) at the 5-year follow-up level. Similarly, UB in both groups was also improved over time (*P* < 0.001).

The unmet need at the time of diagnosis is shown in Additional file 4: Table S2. It was found that more IC patients focused on self-care following surgery as compared with OIN patients (*P* = 0.001), and in the themes of involvement in the treatment decision making more OIN patients made their own personal choice of treatment (*P* = 0.020). The Additional file 5: Table S3 presents the details of unmet needs following surgery, showing that IC patients had less difficulty with urine leakage (*P* = 0.001) and received more help from their family members or friends (*P* = 0.004). The Additional file 6: Table S4 shows the details of unmet needs following surgery during survivorship, indicating that IC patients had more limited physical and social activities (*P* < 0.001), although the OIN patients also had some worries about the future (*P* = 0.011).

## Discussion

There are still some controversies over whether OIN is the most optimal form of UD after RC in terms of HRQoL. Although some studies [11–13] reported that OIN could provide marginally better HRQoL scores than IC, other studies [14–16] argued that there was no significant difference between them. However, there is significant selection bias in these studies in comparing HRQoL between OIN and IC patients, which may cause differences in the preoperative status between these studies. In addition, most of these studies neglected the disease-specific QoL in comparing HRQoL outcomes between the two UDs. For these reasons, we performed a match pair analysis in the present study. Based on the similar baseline characteristics, we evaluated the HRQoL from two aspects at regular intervals postoperatively: one is the change of body image change by using the BIS questionnaire [8], and the other is the disease-specific QoL by using BCI questionnaire [9]. To further understand the details of cancer trajectory, we also presented intermediate profiles by Interview Guide (IG) [10] about the unmet need information. It was found that OIN patients were better in body image, and IC patients were better in UF and UB at the one-year follow-up level. Surprisingly, there was no significant difference in BIS and UB between the two groups at the long-term follow-up level, although UF remained a problem in OIN patients. We also found some differences in unmet need at each time point of the illness trajectory.

It was found in our study that BIS in OIN patients was superior to that in IC patients within the one-year follow-up period. However, a previous study [17] showed no significant difference in BIS, and in their longitudinal model they found that age was correlated with the BIS, suggesting that older patients had slightly better scores. To overcome this shortage, we also performed an MPA in our study in term of age, gender and ASA, score so that the baseline characteristics of these two groups were more comparable. It is worth to mention that there was no significant difference in BIS between the two groups at the long-term follow-up level (*P* = 0.1136), which may be directly related to the positive effect of time, as was the case with that reported in previous studies [17–19], although it needs a relatively long time.

In addition, OIN patients had poorer UF than IC patients, which is consistent with the conclusion of Gilbert et al. [9] who originally designed the BCI questionnaire related to UF, saying that OIN patients had more voiding problems as compared with IC patients. The long-term follow-up results obtained at 12, 24 and 36 months showed that urination always remained a problem in OIN patients, but it tended to be stable after 12 months, which is similar to the conclusion of Hedgepeth et al. [17]. It is generally considered that urinary leakage in OIN patients may result from the loss of reflex micturition and injury to the urethral sphincter. In order to reduce the adverse effects due to urinary dysfunction, patients need to form a new habit of urination and on the other hand the doctor’s instructions about functional training such as Kegel Exercise may help to reinforce the function of the urethral sphincter of patients. As shown in Fig. 2a, the UF was improved markedly at 12 months. However, IC patients had to face the problem of peristomal urinary leak from the pouch, which can only be overcome by good self-care or with the assistance of care providers. We also found that this problem was improved satisfactorily after 12 months. Figure 2b shows that OIN patients had more UB than did IC patients within a year, while there was no significant difference between the two groups in a longer follow-up duration. However, Hedgepeth et al. [17] reported no significant difference in UB between OIN and IC groups, probably because of the difference in baseline UB between the two groups. We consider that poorer UB within a year in OIN group may be due to the UF, because our long-term follow-up observation showed that UB was improved with the improved UF (Fig. 2b). Moreover, UB did not deteriorate significantly compared with that before surgery in IC patients. To some extent, it may be due to the fact that patients always had the UB at the diagnostic phase with the anxiety of caring about malignant tumor for the occurrence of hematuria and urine frequency. Most postoperative patients were bothered by problems of peristomal urinary leakage, foul urine odor and wearing of urine ostomy pouches, which can be improved or adapt. Based on previous studies and our present study, neither of the two UDs shows absolute advantage. Therefore, in the clinical practice, we not only need to make an optimal decision upon surgical options, but also make every endeavor to improve the postoperative HRQoL of special patients.

The measures that we used to assess HRQoL in patients with muscle invasive bladder cancer (MIBC) were unable to distinguish between health related problems and the patient desire or need to receive professional attention or care for these problems. it was imperative to identify and address the unmet needs of patients with MIBC at each time point of the illness trajectory. To optimize the quality of provided health care and gain more comprehensive understanding about potential changes in patient needs and challenges, the responsible physician may be more helpful to the patients to achieve better quality of life. Firstly, more IC patients were concerned about their postoperative self-care problems during the diagnostic phase, presumably because of the apprehension about the postoperative use of urine ostomy pouches. OIN patients without the above worries can make an autonomous choice to live a normal life as ordinary people. Secondly, at the time of following after surgery, OIN patients met with more problems with urine leakage during the short-term postoperative phase, which is consistent with the conclusion of previous studies [17]. However, most of these patients did not seek help from their family members or other care providers, suggesting that urine leakage did not bring them intolerable inconvenience. Thirdly, we found that IC patients had mild physical and social relationship problems during the long-term follow-up phase, which is consistent with a previous perspective study [11]. These problems may be related to the use or leakage of urinary ostomy pouches.

The present study has some limitations. Firstly, the information about the unmet needs in each patient was collected retrospectively, which may run potential risk of recall bias. Secondly, the number of female patients with bladder cancer is relatively small, accounting for about 10 % of all bladder cancer cases in the Chinese population [20, 21]. Thirdly, as nerve-sparing RC was not performed in our included patients, sexual life was lost completely in part of these patients. But as most of our patients were too shy to talk about their sexual life to doctors and other unfamiliar relations, it was difficult for us to make an evaluation on this problem. Fourthly, the number of OIN patients is relatively small. One reason is due to the preference of surgery on the part of the doctor, and the other is due to the consideration on the part of the patient. Fifthly, there is a deviation in our groups’ complication rates according to previous studies, two reasons may affect the complication rate in our study: (1) some patients who had high-grade complications were unwilling to participate in our follow-up questionnaire; (2) some high-grade complications related to radical cystectomy were avoided successfully due to the skilled surgical technique and rich experience of our department.

## Conclusion

BIS scores were significantly better in OIN patients during the short-term (<1 year) follow-up period, while there was no significant difference between IC and OIN groups during the long-term (>1 year) follow-up periods. Urine function remains a problem in OIN patients as compared with IC patients during both short- and long-term follow-up periods. Due attention should be paid to some particular unmet needs in individual patients in managing the two UD modalities.

## Additional files

Additional file 1: Table S5. Peri-operative outcomes. (DOCX 15 kb) Additional file 2: Table S6. Continence outcomes following OIN. (DOCX 15 kb) Additional file 3: Table S1. Demographics and clinical characteristics data before matched pair analysis. (DOCX 19 kb) Additional file 4: Table S2. Unmet informational and supportive care needs at the time of diagnosis. (DOCX 19 kb) Additional file 5: Table S3. Unmet informational and supportive care needs following surgery. (DOCX 18 kb) Additional file 6: Table S4. Unmet informational and support needs during survivorship. (DOCX 18 kb)

## Abbreviations

ASA: American society of anesthesiologists
BCI: Bladder-specific bladder cancer index
BMI: Body mass index
BSI: European organization for research and treatment of cancer body image scale
CUD: Continent urinary diversion
EBL: Estimated blood loss
EORTC-QLQ-C30: European organization for research and treatment of cancer quality-of-life core questionnaire
FACT-G: Functional assessment of cancer therapy-general
HRQoL: Health-related quality-of-life
IC: Ileal conduit
LRC: Laparoscopic radical cystectomy
MIBC: Muscle invasive bladder cancer
MPA: Matched pair analysis
OIN: Ileal neobladder
OT: Operative time
PLND: Pelvic lymph node dissection
RC: Radical cystectomy
UB: Urinary bother
UF: Urinary function
UCB: Urothelial carcinoma of the bladder
