# Supplementary material for: Quality-of-life outcomes and unmet needs between ileal conduit and orthotopic ileal neobladder after radical cystectomy in a Chinese population: a 2-to-1 matched-pair analysis
Source: BMC Urol. 2015 Nov 27;15:117. doi: 10.1186/s12894-015-0113-7 (PMC4662020; doi:10.1186/s12894-015-0113-7)
Supplement: Additional file 1: Table S5. — Peri-operative outcomes. (DOCX 15 kb) [file 12894_2015_113_MOESM1_ESM.docx]

**Table S5. Peri-operative outcomes**

|  | OIN(89) | IC(205) |
| --- | --- | --- |
| Operation time, median (range), min | 346 (210-613) | 297 (180-600) |
| Blood loss, median(range),ml | 231 (50-1000) | 191 (50-1000) |
| Transfusion rate, N (%) | 16 (18.1) | 45 (21.9) |
| No. of lymph nodes removed, mean(range) | 14 (6-47) | 12 (4-34) |
| Perioperative mortality, No | 0 | 0 |
