# Supplementary material for: Quality-of-life outcomes and unmet needs between ileal conduit and orthotopic ileal neobladder after radical cystectomy in a Chinese population: a 2-to-1 matched-pair analysis
Source: BMC Urol. 2015 Nov 27;15:117. doi: 10.1186/s12894-015-0113-7 (PMC4662020; doi:10.1186/s12894-015-0113-7)
Supplement: Additional file 2: Table S6. — Continence outcomes following OIN. (DOCX 15 kb) [file 12894_2015_113_MOESM2_ESM.docx]

|  | OIN (89) |
| --- | --- |
| Daytime continence rates | 76 (85.4) |
| Nighttime continence rates | 65 (73.0) |
| ISC rates | 2 (2.2) |
| Maximum Neobladder volume (mL) | 346±31 |
| Postvoid residual (mL) | 28±14 |

**Table S6. Continence outcomes following OIN**
