# Supplementary material for: Quality-of-life outcomes and unmet needs between ileal conduit and orthotopic ileal neobladder after radical cystectomy in a Chinese population: a 2-to-1 matched-pair analysis
Source: BMC Urol. 2015 Nov 27;15:117. doi: 10.1186/s12894-015-0113-7 (PMC4662020; doi:10.1186/s12894-015-0113-7)
Supplement: Additional file 3: Table S1. — Demographics and clinical characteristics data before matched pair analysis. (DOCX 19 kb) [file 12894_2015_113_MOESM3_ESM.docx]

**Table S1. Demographics and clinical characteristics data before matched pair analysis**

| Characteristic | Total 294 | | *P* |
| --- | --- | --- | --- |
|  | OIN (89) | IC (205) |  |
| Mean Age, yr (range) | 61 (53-68) | 66(59-73) | 0.0002^&^ |
| Sex ratio (M/F) | 80/9 | 179/26 | 0.532^*^ |
| Mean BMI, kg/m^2^ (range) | 22.3 (19.3-25.6) | 21.4 (18.1-24.4) | 0.061^&^ |
| ASA class, N (%) |  |  | 0.504^&^ |
| 1 | 17 (19.1) | 28 (13.7) |  |
| 2 | 54 (60.7) | 136 (66.3) |  |
| 3 | 18 (20.2) | 41 (20.0) |  |
| Smoking history, N (%) | 24 (27.0) | 43 (21.0) | 0.261^*^ |
| Previous abdominal or pelvic surgery, N (%) | 13 (14.6) | 22 (10.7) | 0.346^*^ |
| Comorbidities, N (%) |  |  |  |
| Cardiovascular disease | 6 (6.7) | 11(5.4) | 0.642^*^ |
| Pulmonary disease | 13 (14.6) | 31 (15.1) | 0.909^*^ |
| Hypertension | 24 (27.0) | 64 (31.2) | 0.464^*^ |
| Diabetes | 15 (16.9) | 42 (20.5) | 0.469^*^ |
| Histological type, N (%) |  |  | 0.354^**^ |
| Pure TCC | 82 (92.1) | 196 (95.6) |  |
| Other pathology | 7 (7.9) | 9 (4.4) |  |
| Histological grade, N (%) |  |  | 0.015^*^ |
| Grade 1 and Grade 2 | 26 (29.2) | 34 (16.6) |  |
| Grade 3 | 58 (65.7) | 157 (76.6) |  |
| Pathologic T stage, N (%) |  |  | 0.444^&^ |
| Organ-confined: ≤pT2, pN0 | 74 (83.1) | 164 (80.0) |  |
| Non-organ-confined: pT3-pT4, pN0 | 13 (14.6) | 27 (13.2) |  |
| Lymph node-positive: pN+ | 2 (2.2) | 14 (6.8) |  |
| Highest grade of complication, N (%) |  |  | 0.010^&^ |
| I | 11 (12.4) | 27 (13.2) |  |
| II | 10 (11.2) | 2 (1.0) |  |
| III | 8 (9.0) | 4 (2.0) |  |
| IV | 0 | 2 (1.0) |  |
| V | 0 | 0 |  |

^&^: Wilcoxon rank-sum test; ^*^: Chi-Square; **^**^:** Pearson chi-squared test with continuity correction
