# Supplementary material for: Quality-of-life outcomes and unmet needs between ileal conduit and orthotopic ileal neobladder after radical cystectomy in a Chinese population: a 2-to-1 matched-pair analysis
Source: BMC Urol. 2015 Nov 27;15:117. doi: 10.1186/s12894-015-0113-7 (PMC4662020; doi:10.1186/s12894-015-0113-7)
Supplement: Additional file 4: Table S2. — Unmet informational and supportive care needs at the time of diagnosis. (DOCX 19 kb) [file 12894_2015_113_MOESM4_ESM.docx]

**Table S2: Unmet informational and supportive care needs at the time of diagnosis**

| Unmet Needs | OIN (39) | IC (78) | P |
| --- | --- | --- | --- |
|  | N (%) | N (%) |  |
| Information believe to be useful but not offered or fully discussed by the physician  (Themes) |  |  |  |
| Treatment options and side effects | 34 (87.2) | 71 (91.0) | 0.747** |
| Recovery rate | 39 (100.0) | 77 (98.7) | 1.000** |
| Medical bill | 27 (69.2) | 58 (74.4) | 0.558* |
| Self-care following surgery | 18 (46.2) | 41 (52.6) | 0.513* |
| Preparation for surgery | 35 (89.7) | 61 (78.2) | 0.125* |
| Change in sexual function | 14 (35.9) | 23 (29.5) | 0.482* |
| Searched for information about treatment  (Themes) |  |  |  |
| Talked to other patients | 11 (28.2) | 28 (35.9) | 0.405* |
| Obtain from doctor | 23 (59.0) | 51 (65.4) | 0.498* |
| Searched the internet | 5 (12.8) | 9 (11.5) | 1.000** |
| Type of information about treatment options and treatment side effects  (Themes) |  |  |  |
| Cancer control and recovery rate | 29 (74.3) | 62 (79.5) | 0.529* |
| Satisfaction with treatment outcomes | 21 (53.8) | 35 (44.9) | 0.360* |
| Post-surgical self-care | 14 (35.9) | 53 (67.9) | 0.001* |
| Involvement in the treatment decision making  (Themes) |  |  |  |
| Made their decision based on physicians’ recommendation | 25/28 (8.3) | 52/58 (89.7) | 1.000* |
| Made their own personal choice of treatment | 16/28 (57.1) | 18/58 (31.0) | 0.020* |
| Made treatment decision after considering family members recommendations | 13/28 (46.4) | 19/58 (32.8) | 0.219* |
| Worries about treatment  (Themes) |  |  |  |
| Worries about survival | 32 (82.1) | 67 (85.9) | 0.587* |
| Worries about the surgical outcomes | 27 (69.2) | 58 (74.4) | 0.558* |
| Worries about treatment side effects/Worries about changes in sexual function | 19 (48.7) | 34 (43.6) | 0.599* |
| Emotional distress  (Themes) |  |  |  |
| Felt depressed | 9 (23.1) | 24 (30.8) | 0.383* |
| Had negative emotions | 14 (35.9) | 29 (37.2) | 0.892* |
| Felt shocked and scared | 26 (66.7) | 55 (70.5) | 0.671* |
| Felt angry | 21 (53.8) | 47 (60.3) | 0.508* |

*: Chi-Square; ****:** Pearson chi-squared test with continuity correction
