# Supplementary material for: Quality-of-life outcomes and unmet needs between ileal conduit and orthotopic ileal neobladder after radical cystectomy in a Chinese population: a 2-to-1 matched-pair analysis
Source: BMC Urol. 2015 Nov 27;15:117. doi: 10.1186/s12894-015-0113-7 (PMC4662020; doi:10.1186/s12894-015-0113-7)
Supplement: Additional file 5: Table S3. — Unmet informational and supportive care needs following surgery. (DOCX 18 kb) [file 12894_2015_113_MOESM5_ESM.docx]

**Table S3: Unmet informational and supportive care needs following surgery**

| Unmet Needs | OIN (39) | IC (78) | P |
| --- | --- | --- | --- |
|  | N (%) | N (%) |  |
| Difficulty related to recovery |  |  |  |
| Difficulty related to physical recovery | 6 (15.4) | 17 (21.8) | 0.411* |
| Difficulty related to emotional recovery | 11 (28.2) | 19 (24.4) | 0.653* |
| Difficulties with bowel function | 3 (7.7) | 7 (9.0) | 1.000** |
| Had difficulty after surgery from changes in urinary function |  |  |  |
| Had little or no control of urine | 19 (48.7) | NA |  |
| Difficulty with urine leakage | 27 (69.2) | 28 (35.9) | 0.001* |
| Bother with incontinence | 2 (5.1) | NA |  |
| Bother with odor | 13 (33.3) | 22 (28.2) | 0.568* |
| Had difficulty related to use of stomal |  |  |  |
| Difficulty with the use of stomal appliances | NA | 51 (65.4) | - |
| Had allergic reaction to stomal applicances | NA | 44 (56.4) | - |
| Difficulty with changing the stomal bag when away from home | NA | 38 (48.7) | - |
| Had difficulty related to neobladder care |  |  |  |
| Difficulty using public restrooms | 12 (30.8) | NA | - |
| Difficulty with irrigation | 28 (71.9) | NA | - |
| Difficulty because of infection | 17 (43.6) | NA | - |
| Difficulty urinating with a neobladder | 4 (10.3) | NA | - |
| Spouse/partner helped with stomal care |  |  |  |
| Helped with changing the stomal bag | NA | 53 (67.9) | - |
| Help with ordering stomal appliances | NA | 36 (46.2) | - |
| Provided needed information about stomal care | NA | 49 (62.8) | - |
| Spouse/partner helped with other things related to treatment |  |  |  |
| Helped holding and cleaning appliances | 17 (43.6) | 36 (46.2) | 0.793* |
| Helped with changing the wet bed-sheets | 14 (35.9) | 24 (30.8) | 0.577* |
| Helped with putting cloth on | 4 (10.3) | 7 (9.0) | 1.000** |
| Patient relied on him/herself in post-treatment self-care |  |  |  |
| Did not receive help from family members or friends with self-care after treatment | 33 (84.6) | 45 (57.7) | 0.004* |

*: Chi-Square; ****:** Pearson chi-squared test with continuity correction
