# Supplementary material for: Quality-of-life outcomes and unmet needs between ileal conduit and orthotopic ileal neobladder after radical cystectomy in a Chinese population: a 2-to-1 matched-pair analysis
Source: BMC Urol. 2015 Nov 27;15:117. doi: 10.1186/s12894-015-0113-7 (PMC4662020; doi:10.1186/s12894-015-0113-7)
Supplement: Additional file 6: Table S4. — Unmet informational and support needs during survivorship. (DOCX 18 kb) [file 12894_2015_113_MOESM6_ESM.docx]

**Table S4: Unmet informational and support needs during survivorship**

| Unmet Needs | OIN (39) | IC (78) | P |
| --- | --- | --- | --- |
|  | N (%) | N (%) |  |
| Difficulties in daily living |  |  |  |
| Limited physical and social activities | 4 (10.3) | 34 (43.6) | ＜0.001* |
| Could not continue working | 14 (35.9) | 37 (47.4) | 0.235* |
| Stayed closer to home | 26 (66.7) | 54 (69.2) | 0.779* |
| Always on the lookout for public restrooms when away from home | 9 (23.1) | 11 (14.1) | 0.224* |
| Changed clothing style | 7 (17.9) | 20 (25.6) | 0.352* |
| Emotional distress after the treatment |  |  |  |
| Felt depressed after surgery | 12 (30.8) | 27 (34.6) | 0.677* |
| Had emotional distressed after surgery | 8 (20.5) | 19 (24.4) | 0.642* |
| Felt sad | 5 (12.8) | 19 (24.4) | 0.145* |
| Not feeling “normal” | 28 (71.8) | 53 (67.9) | 0.671* |
| Received help with emotional distress |  |  |  |
| Took medication for depression | 2 (5.1) | 1 (1.3) | 0.535** |
| Had psychological counseling | 6 (15.4) | 5 (6.4) | 0.218** |
| Turned to religion and spirituality to cope with depression | 0 | 0 | - |
| Worried about the future with regards to the treatment they received |  |  |  |
| Worries about the future | 16 (41.0) | 15 (19.2) | 0.011* |
| Worries about cancer recurrence | 23 (59.0) | 54 (69.2) | 0.270* |
| Worries about self-caring in the future | 9 (23.1) | 23 (29.5) | 0.463* |
| Worries about urine control in the future | 21 (53.8) | NA | - |

*: Chi-Square; ****:** Pearson chi-squared test with continuity correction
